# Investigations on Caesium Dispersion and Molybdenum Coating on SPIDER Components

**DOI:** 10.3390/ma16010206

**Published:** 2022-12-26

**Authors:** Valentina Candela, Caterina Cavallini, Claudia Gasparrini, Lidia Armelao, Valeria Candeloro, Mauro Dalla Palma, Michele Fadone, Diego Marcuzzi, Mauro Pavei, Adriano Pepato, Basile Pouradier Duteil, Marzio Rancan, Andrea Rizzolo, Emanuele Sartori, Beatrice Segalini, Gianluigi Serianni, Monica Spolaore, Federico Zorzi, Piergiorgio Sonato

**Affiliations:** 1 Centro Ricerche Fusione, Università degli Studi di Padova, Corso Stati Uniti 4, 35127 Padova, Italy; 2 Istituto Nazionale di Fisica Nucleare (INFN)—Sezione di Padova, Via Marzolo 8, 35131 Padova, Italy; 3 Consorzio RFX, CNR, ENEA, INFN, Università di Padova, Acciaierie Venete SpA, Corso Stati Uniti 4, 35127 Padova, Italy; 4 Department of Materials & Centre for Nuclear Engineering, Imperial College London, London SW7 2AZ, UK; 5 Department of Chemical Sciences, University of Padova, Via F. Marzolo 1, 35131 Padova, Italy; 6 Department of Chemical Sciences and Materials Technologies (DSCTM), National Research Council (CNR), Piazzale A. Moro 7, 00185 Roma, Italy; 7 CNR—Istituto per la Scienza e Tecnologia dei Plasmi, 35127 Padova, Italy; 8 Swiss Plasma Center (SPC), Ecole Polytechnique Fédérale de Lausanne (EPFL), CH-1015 Lausanne, Switzerland; 9 Institute of Condensed Matter Chemistry and Technologies for Energy (ICMATE), National Research Council (CNR), via F. Marzolo 1, 35131 Padova, Italy; 10 Dipartimento di Ingegneria Industriale, Università degli Studi di Padova, Via Gradenigo 6, 35131 Padova, Italy; 11 Centro di Analisi e Servizi Per la Certificazione (CEASC), University of Padua, 35121 Padua, Italy

**Keywords:** SPIDER, ITER, caesium evaporation, NBI, caesium deposits

## Abstract

SPIDER is the 100 keV full-size Negative Ion Source prototype of the ITER Neutral Beam Injector, operating at Consorzio RFX in Padova, Italy. The largest Negative Ion Source in the world, SPIDER generates an RF driven plasma from which Deuterium or Hydrogen negative ions are produced and extracted. At the end of 2021, a scheduled long-term shutdown started to introduce major modifications and improvements aiming to solve issues and drawbacks identified during the first three years of SPIDER operations. The first action of the shutdown period was the disassembly and characterization of the SPIDER beam source after removal from the vacuum vessel and its placement inside the clean room. Each component was carefully assessed and catalogued, following a documented procedure. Some source components, i.e., the Plasma Grid, Extraction Grid and Bias Plate, revealed the presence of different and non-uniform red, white and green coatings that might be correlated to back-streaming positive ions impinging on grid surfaces, electrical discharges and caesium evaporation. Thus, several analyses have been carried out to understand the nature of such coatings, with the study still ongoing. The evidence of caesium evaporation and deposition on molybdenum-coated SPIDER components, such as the formation of oxides and hydroxides, is demonstrated through surface characterization analyses with the use of the Scanning Electron Microscope (SEM), X-ray Diffraction (XRD) and X-ray Photoelectron Spectroscopy (XPS).

## 1. Introduction

The Source for the Production of Ion of Deuterium Extracted from Rf plasma (SPIDER) is located in Padova, Italy, at Consorzio RFX. SPIDER is a full-size prototype of the radio frequency negative ion source for the International Thermonuclear Experimental Reactor (ITER) neutral beam injector (NBI) equipped with a 100 keV accelerator.

SPIDER serves to evaluate and optimize the performance of the ITER ion sources. It is designed to extract and accelerate H^−^ and D^−^ up to 100 keV with a maximum extraction current density of 355 [A/m^2^] in hydrogen and 285 [A/m^2^] in deuterium, a beam source filling pressure of 0.3 Pa and pulse length of 3600 s [1,2].

**Figure 1:**
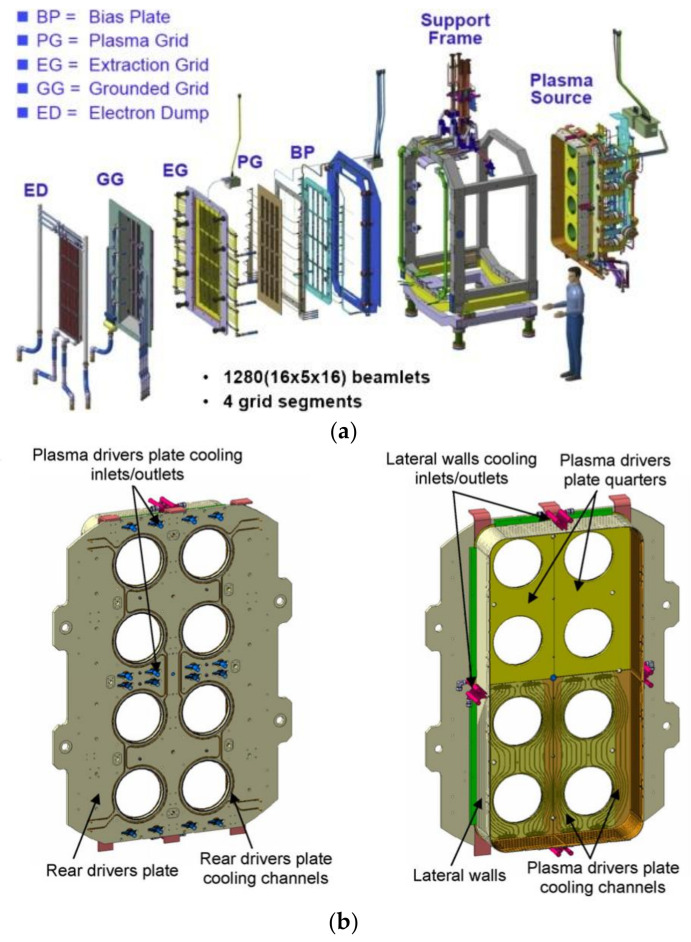

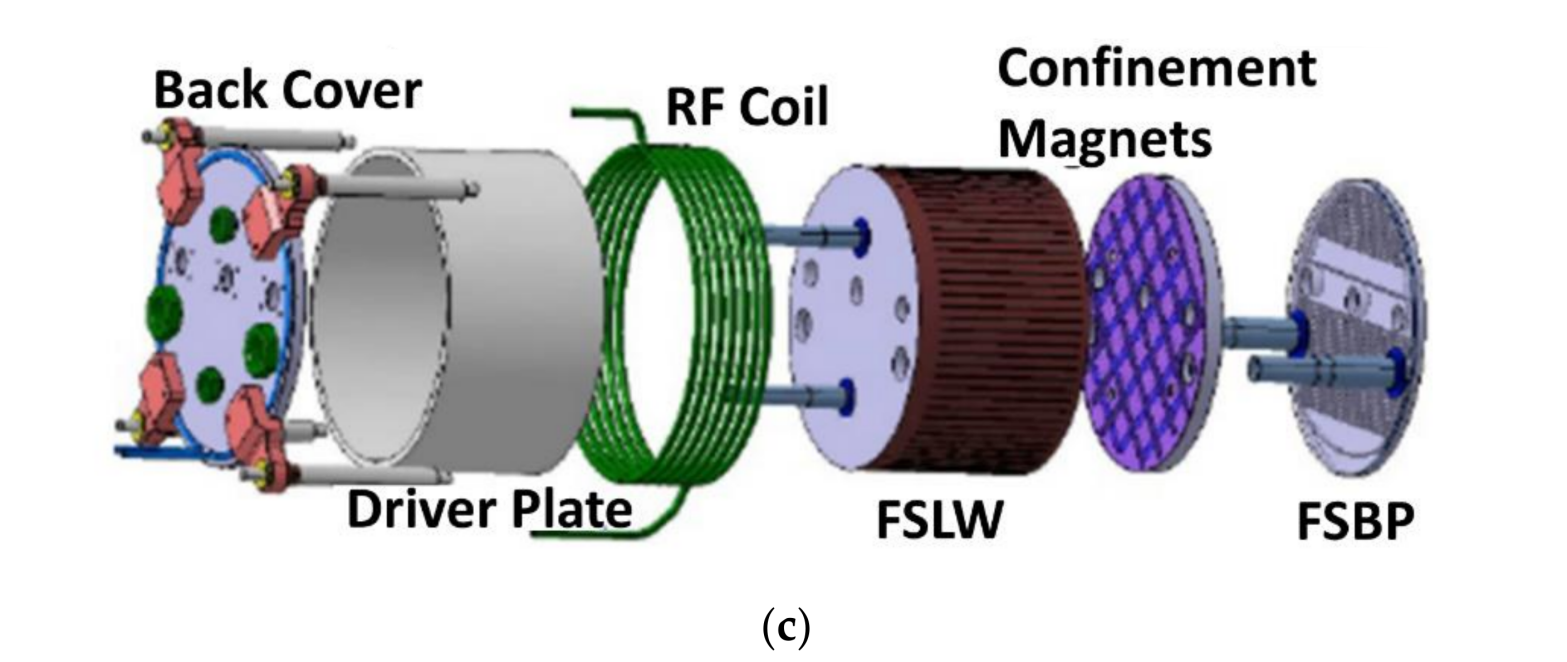
(**a**) Exploded view of SPIDER Beam Source; (**b**) Drivers Plate rear view on the left and front view on the right; (**c**) exploded view of one driver [3,5].

The SPIDER extraction and acceleration system is composed of three grids: the Plasma Grid (PG), Extraction Grid (EG) and Grounded Grid (GG) plus a Bias Plate (BP) (Figure 1a). The BP, placed upstream of the PG, reduces the co-extracted electron current. Each grid features 1280 apertures, through which the ion beamlets are extracted from the ion source and accelerated up to 100 keV energy. All the grids are constructed by electro-deposition of pure copper onto a copper base plate. The BP and PG are covered by a molybdenum layer, a refractory metal with a low sputtering yield [3,4], of about 100 µm thickness to prevent copper sputtering due to the impact of the plasma. Copper sputtering is highly undesirable since Cu^+^ ions are accelerated back to the source, leading to further sputtering.

SPIDER plasma is generated in 8 Radio Frequency (RF) drivers installed on the Rear Driver Plate (RDP), a stainless-steel plate directly connected to a second CuCrZr plate, the Plasma Driver Plate (PDP), which is directly exposed to the plasma (Figure 1b). Each driver is equipped with an alumina case and protected from plasma by a copper Faraday Shield Lateral Wall (FSLW). At the back of each driver, the CuCrZr Faraday Shield Back Plate (FSBP) is located (Figure 1c) [3,5].

Caesium is evaporated into the source through three caesium ovens installed on the PDP to increase the negative ion density in the proximity of the extraction region. The Cs deposit on the Plasma Grid (PG) lowers the work function, promoting negative ion surface production. It is experimentally observed that optimum Cs coverage on tungsten (W) or molybdenum (Mo) surfaces can achieve very low work functions in the range of 1.4–1.6 eV [6]. During negative ion source operation, the Cs monolayer is continuously renewed, with the caesiated surfaces maintained at 150 °C to keep the work function as low as possible and preserve the balance between Cs evaporation and deposition rates [7,8].

SPIDER started to operate in 2018 and, after more than three years, a major shutdown started in December 2021 to carry out improvements and modifications [2].

In December 2021, before the shutdown, a water leak occurred in one of the eight FSLWs, resulting in a water spray that wetted the lower part of the source, including the BP and PG. Soon after, the long shutdown started and the beam source was removed from the vacuum vessel, disassembled and characterized. The source exhibits evidence of several phenomena that occurred during SPIDER operation: non-uniform distribution of caesium across the plasma electrode (PG and BP), back-streaming positive ions impinging on the rear side of the source case lateral wall, which embeds the source, and electrical discharges outside and inside the source. During the dismantling, the presence of compounds on the surface of the components was observed, likely due to reactions between the Cs and Mo coatings and water. This article presents the study and analyses of the morphological structure, chemical composition and distribution of such compounds found on SPIDER grid surfaces. Collected samples were characterized by scanning electron microscopy (SEM), X-ray diffraction (XRD) and X-ray photoelectron spectroscopy (XPS) confirming a non-uniform distribution of evaporated caesium on the PG and a direct effect of the water leak on caesium deposition.

## 2. Materials and Methods

**Figure 2:**
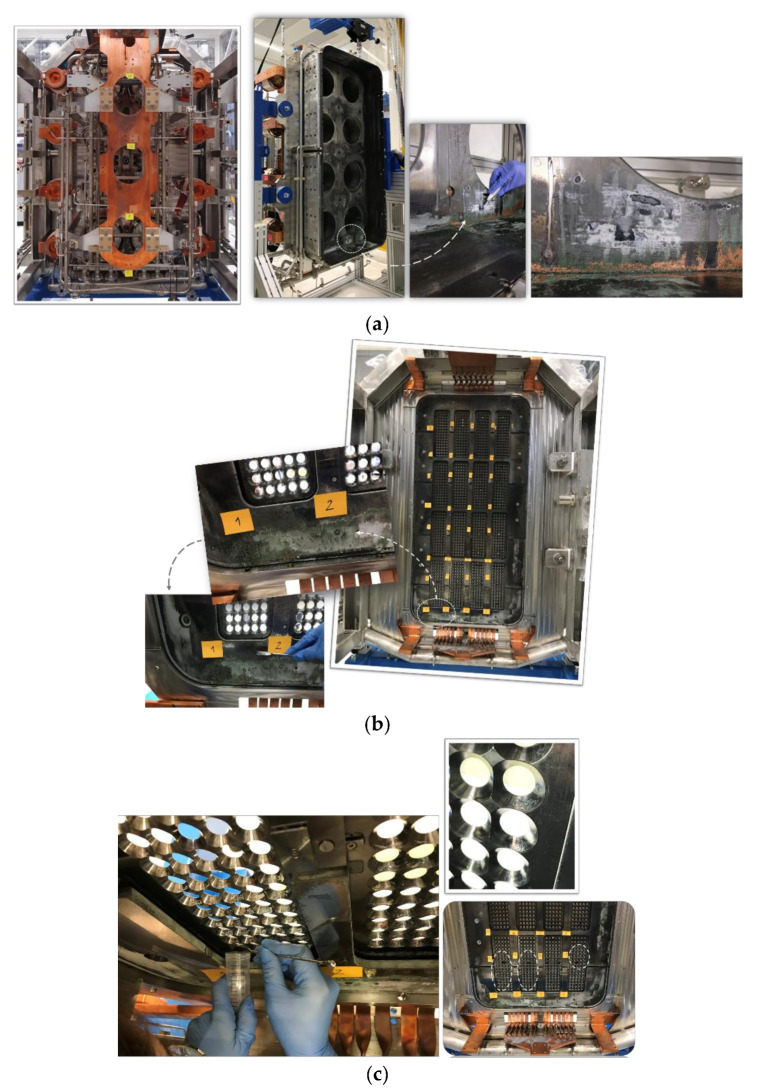
Compounds taken from (**a**) Driver Plate (the Rear Driver Plate is on the left, while the Plasma Driver Plate can be seen on the right); (**b**) Bias Plate; and (**c**) Plasma Grid.

Several analysis methods were adopted to understand the chemical nature of the compounds produced on the PG, BP and PDP (Figure 2). To better examine what happened to the source during operation and after the water leak, several specimens, listed in Table 1, were taken from different components of the SPIDER source. Compounds were taken by scratching the coating away with a flat stainless-steel spatula with the powders stored in vials. The lower part of the source was the investigated region, where the heterogeneity of the coating was visible to the naked eye.

**Figure 3:**
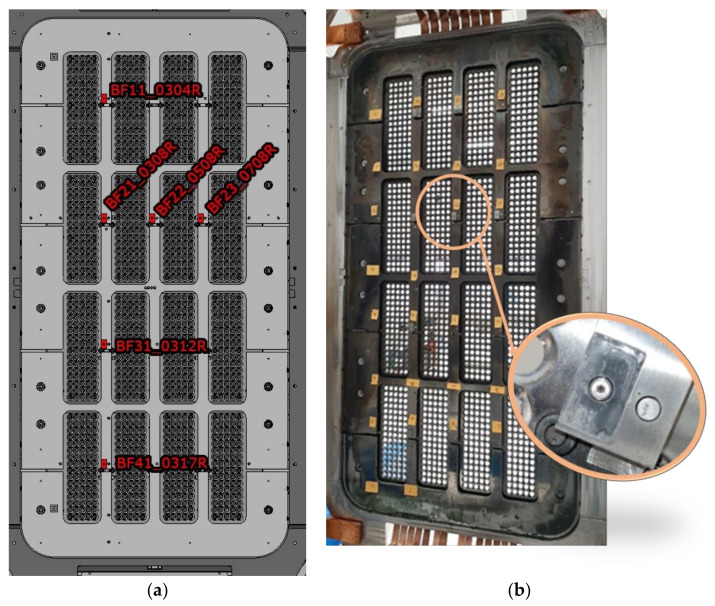
(**a**) BF Probes name and (**b**) position on BP.

In addition, some bulk Mo targets, called Bias plate grid Front side probes (BF probes), which are components of the Langmuir probes fastened on the BP for diagnostic purposes, were analyzed in detail. These specimens are representative of the overall PG and BP surfaces since they were placed both in the upper and lower halves of the source. Five out of six BF probes were unscrewed to be analyzed. Their names and distribution are reported in Figure 3. BF31_0312R is the sample that was impossible to remove.

Morphology analyses were carried out using Scanning Electron Microscopy (SEM) at different magnifications. The chemical composition of the samples has been investigated with SEM Energy Dispersion Spectroscopy (SEM-EDS), X-ray Diffraction (XRD), X-ray Photoelectron Spectroscopy (XPS) and Fourier-Transform InfraRed (FTIR) spectroscopy analyses. 

The SEMs used for the study were FEI QUANTA 200 (FEI, Hillsboro, OR, USA) and COXEM EM3AX Plus (COXEM Co., Daejeon, Korea) microscopes. Both SEM microscopes worked with a W-filament source and with an accelerating voltage of 20 kV. Samples were observed without any preparation since, in most cases, the observations have been conducted in low-vacuum mode. The SEMs included an X-ray detector EDAX Element-C2B. The SEM-EDS analyses and mapping elaborations were conducted with a TEAM^TM^ EDS Analyses System (EDAX Inc.—AMETEK Inc., Berwyn, PA, USA) for monitoring the presence of Cs on sample surfaces. 

XRD analyses were conducted on some of the powders and on some of the BF Probes using Panalytical XPert 3 Powder (Malvern Panalytical Ltd., Malvern, UK). The instrument consisted of a Cu-anode X-ray tube and solid-state detector PixCel. Twelve 1 h-long analyses were performed for each specimen.

Sample 4 (Table 1) taken from the area next to one of the drivers was also examined by means of FTIR analyses, since hydrogen cannot be easily detected by the most common chemical investigation methodologies. This analysis was important to establish the presence of hydroxides within the compounds taken from the surfaces of BP and Source Case Lateral Wall. The instrument used was the Fourier Nicolet iS-50 (Thermo Fisher Scientific, Waltham, MA, USA) and the software employed for the elaboration of the spectra was OMNIC^®^ 9.

Furthermore, XPS analyses were performed on different samples with a PerkinElmer Φ 5600-ci spectrometer using Al Kα radiation (1486.6 eV). The sample analysis area was 800 μm in diameter. The standard deviation for the Binding Energies (BEs) values was ±0.2 eV. Further details may be found elsewhere [9,10].

In the end, Gamma spectroscopy was conducted on the BF Probes and a spare electrode to establish the nature of niobium (Nb) detected during SEM-EDS analyses. Since the probes faced the plasma during the source operation, and the original material was pure bulk Mo, spectroscopy investigations were performed to exclude the formation of Nb isotopes from Mo. Indeed, 94Mo is generally present in nature at 9.18% [11] and in some specific cases, can lead to the formation of 91Nb m/g after a no-threshold p/alpha decay reaction.

## 3. Results

### 3.1. Powder Samples from PG, BP and PDP

**Figure 4:**
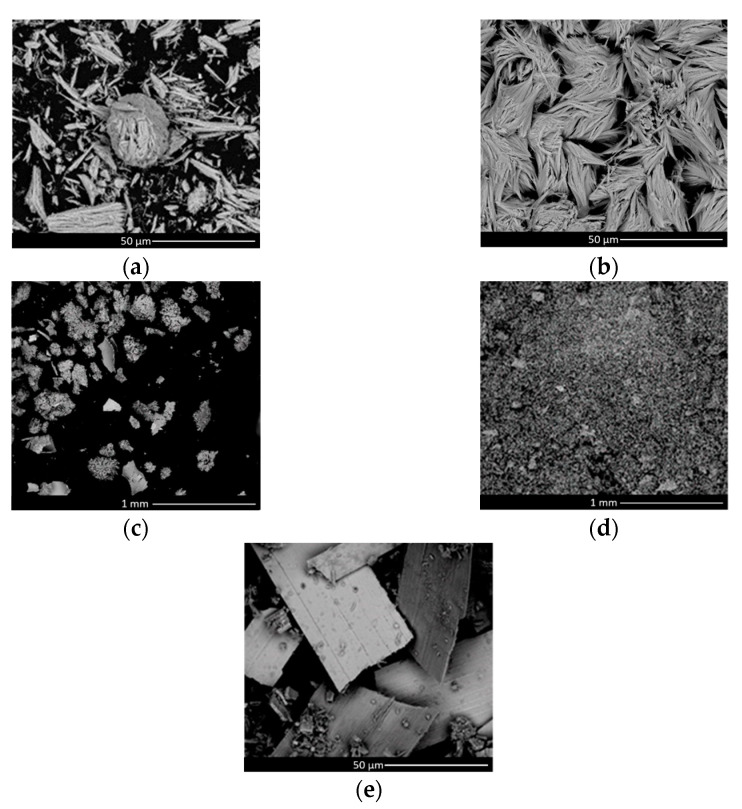
SEM image of samples taken from SPIDER: (**a**) Sample PDP_4, (**b**) Sample PDP_6, (**c**) Sample BP_9 (bottom left) (**d**) Sample BP_10 (bottom right), (**e**) Sample PG_11 (beamlets apertures).

**Figure 5:**
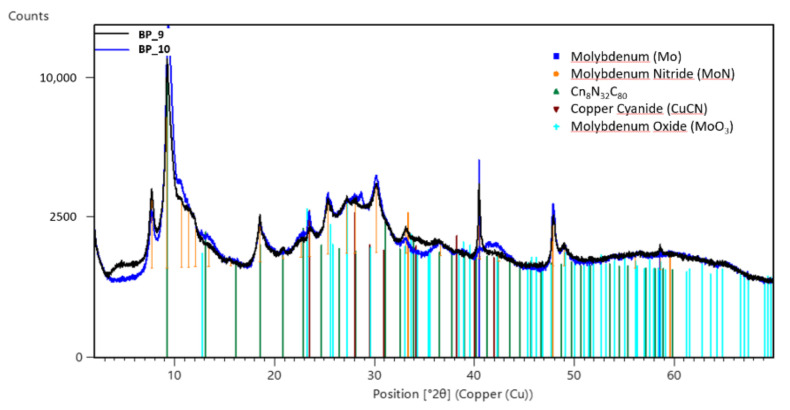
XRD analyses of samples BP_9 and BP_10.

The study and analyses of samples listed in Table 1 are reported in this section. Sample 3 is the only one that is further treated only using the FTIR technique. Analyzed with SEM, all samples showed a different morphology and crystalline structure depending on the sampling region. Samples PDP_4 and PDP_6 (Table 1) were characterized by structures similar to short fibers mainly made of Molybdenum, Copper and Oxygen. On the BP and PG surfaces, collected samples BP_9, BP_10 and PG_11 showed a bulky aspect. Fiber structures were less evident; meanwhile, particles or chips were the dominant shapes (Figure 4). The varying structures might be due to a difference in environmental conditions or a different speed of change. The EDS analyses revealed that all samples had the same chemical elements. The XRD analyses (Figure 5), performed on samples BP_9 and BP_10, showed that all the compounds had an amorphous phase that did not allow an appropriate matching of the pattern’s peaks with the database. Concerning the chemical species, great correspondence with molybdenum oxides and nitrides has been found. The oxides reasonably come from the interaction between the molybdenum layer and water, while nitrides could have been produced due to atmosphere exposure after the source opening.

**Figure 6:**
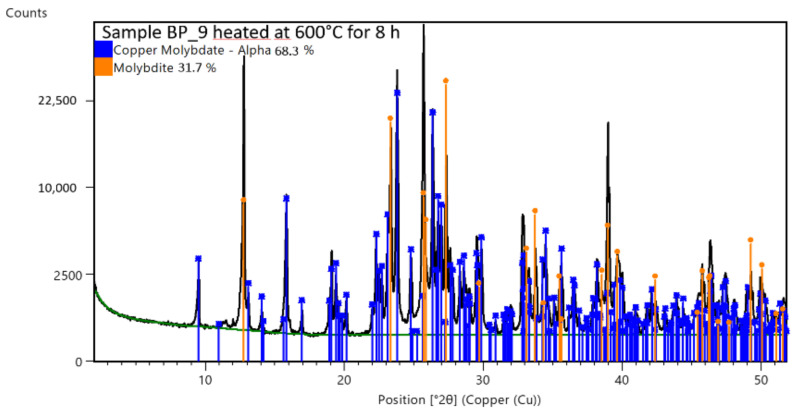
XRD of sample BP_9 after heating at 600 °C for an entire night (about 8 h).

Samples BP_9 and BP_10 were heated to eliminate most of the amorphous phase. The heat treatment consisted of maintaining the powders at 600 °C inside a furnace overnight to convert the hydroxides into oxides and obtain crystalline structures. No protective inert atmosphere was used. XRD analyses were repeated and the pattern revealed well-defined peaks. The data elaboration established that complex oxides of Mo and Cu were the main constituents of the two samples. Figure 6 shows the XRD analysis performed on the heat-treated sample BP_9, with similar results for sample BP_10.

**Figure 7:**
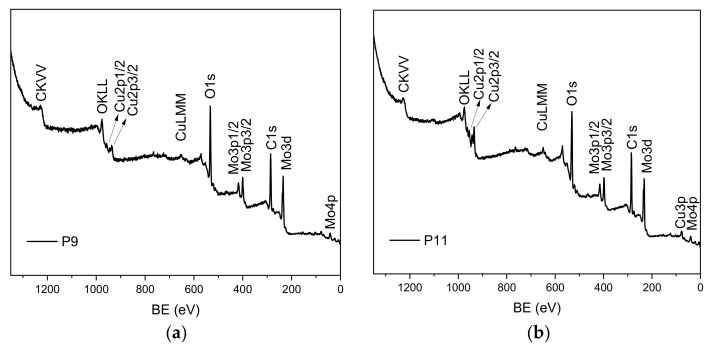
XPS survey analyses of sample BP_9 and sample PG_11. (**a**) XPS Sample BP_9, (**b**) XPS Sample PG_11.

The XPS analyses, reported in Figure 7, agree with the XRD results: powder surfaces mainly contain Mo, Cu, O and adventitious carbon, while the atomic percentages of the detected elements are reported in Table 2. None of the specimens revealed the presence of Cs. This aspect can be explained by considering that water could have washed Cs away from source surfaces. Caesium reacts with water in an exothermal and explosive reaction to give the highly hygroscopic CsOH according to [12]:

(1) $$2\mathrm{Cs}\left(s\right)+2H_{2}O\to \;2\mathrm{CsOH}\left(\mathrm{aq}\right)+H_{2}\left(g\right)$$

### 3.2. BF Probes

**Figure 8:**
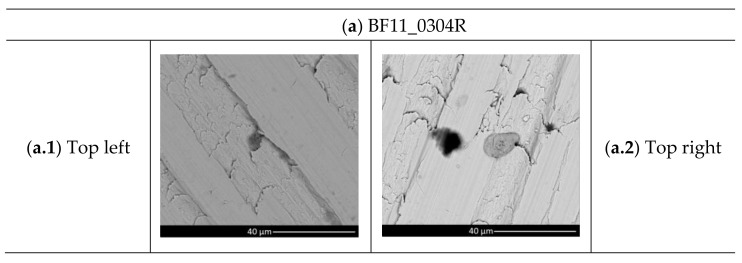

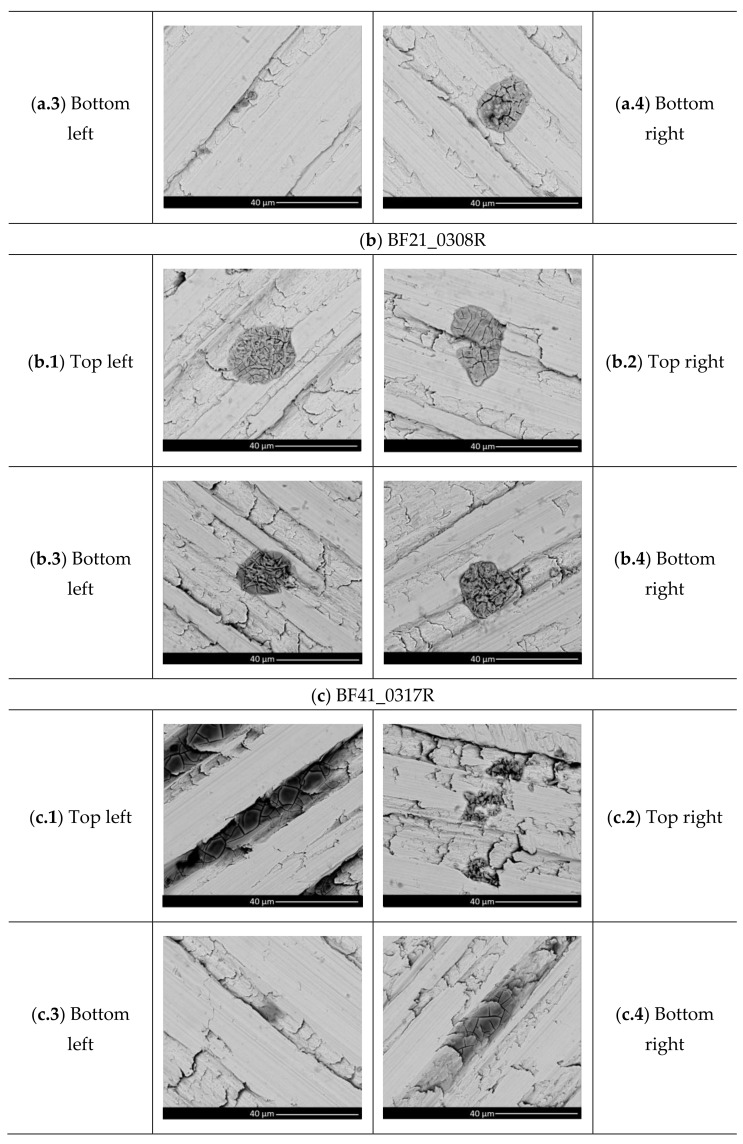
SEM analyses of BF11, BF21 and BF41 probes.

The molybdenum surface targets were investigated by SEM, revealing a non-uniform Cs distribution. As reported in Figure 8, Cs was found on top of them, except for the BF41, which is the one originally placed on the lower BP segment. Cs was distributed in droplets: unfortunately, the optimal monolayer for negative ion production was not observed on probe surfaces. This is the only clear evidence found on all the collected samples of Cs evaporation in the SPIDER source. As an example, Figure 8 reports the SEM images of three out of six BF probes.

It is important to note that BF41 is totally Cs-free, similarly to the powder samples previously analyzed (Table 1). BF41 and the powder samples were all located on and collected from the bottom of the source, which was subjected to the water leak. Therefore, the possible presence of Cs could have been removed by reaction with water Equation (1).

**Figure 9:**
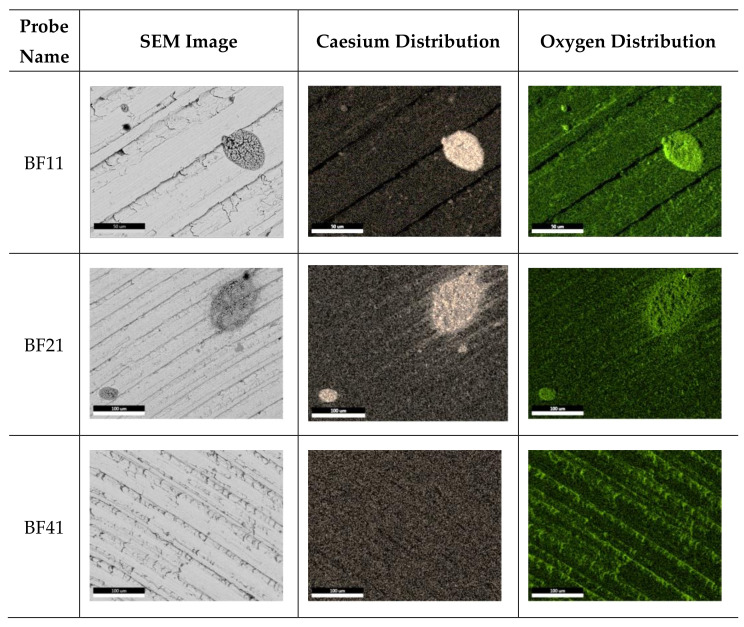
Compositional map of Cs and O on probes BF11, BF21 and BF41.

Figure 9 reports the compositional map of Cs and O on probes BF11, BF21 and BF41. With regard to oxygen, it is clear that the Cs droplets are oxidized. Caesium oxide and hydroxide CsOH, formed due to reaction Equation (1), could have been formed after opening the source. The caesium is mainly located in precise structures, where the brownish points are more concentrated. Only BF21 revealed a dispersion of Cs outside the droplet borders.

The evidence of Cs evaporation in droplets is not well understood. Further investigations need to be carried out to understand if Cs agglomerates were formed during the source opening when Cs was exposed to air and humidity or if the monolayer was never created. From a morphological point of view, caesium is dispersed in circular indented structures. The EDS percentage composition result underlined that oxygen is always strongly combined with caesium: a lower Cs/O ratio compared to Mo/O was observed for all probes. Figure 9 highlights that oxygen is strictly bonded with Cs droplets. Unfortunately, despite an expected increase in oxygen concentration for the bottom probes due to water cleaning the source surfaces, a clear trend in oxygen distribution was not found.

**Figure 10:**
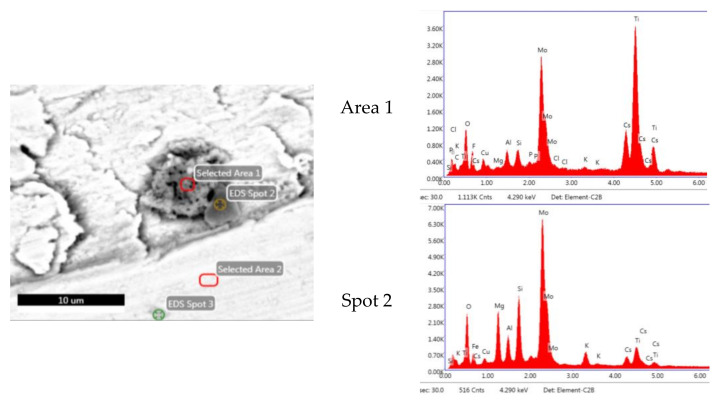
EDS-SEM of structures made of titanium.

The SEM-EDS also detected the presence of titanium, bonded in a porous and squared structure with caesium (Figure 10). The nozzle from which Cs is evaporated is made of TZM alloy and Cs could have stripped some small Ti chips from it. The fact that Ti was only found in these Cs structures, and not alone, supports this theory.

**Figure 11:**
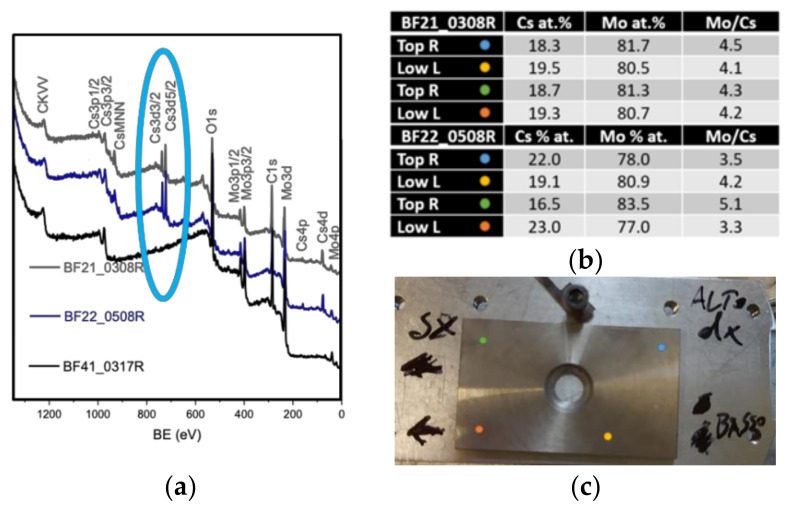
XPS analysis results on BF21, BF22 and BF41. (**a**) XPS surveys, (**b**) chemical composition for samples BF21 and BF22, (**c**) example of BF probe orientation and analyzed spots.

XPS analyses, carried out on BF21, BF22 and BF41 and reported in Figure 11, confirmed that all three BF probes had similar chemical composition but for BF41, where the presence of Cs was not detected. XRD analyses did not give any further information: Cs quantity was too low to be detected with this analysis and the only match found in database for XRD patterns was the one of pure Mo.

### 3.3. FTIR

**Figure 12:**
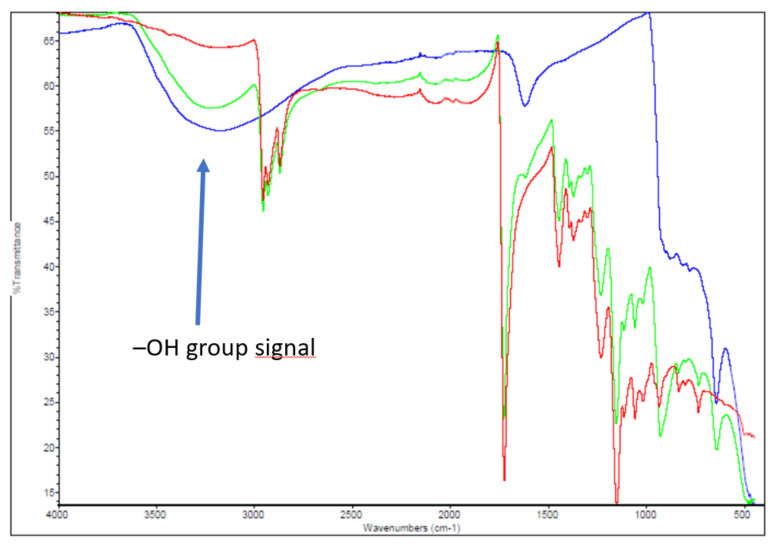
FTIR spectra of sample 3 and sample PDP_4 (Table 1).

In Figure 12, the chart obtained examining the powders removed from the source using FTIR spectroscopy is reported; the red and green spectra are the results of the specimen taken next to driver L4 (Sample PDP_4, Table 1), from which the water leak occurred, while the blue line represents the coatings taken from different parts of the source (Sample 3, Table 1).

Sample n.4, with a flake shape, was analyzed twice; the red line indicates the side exposed to the electron beam, while the green spectrum represents the other side, covered by adhesive applied previously for SEM. In both cases, the spectrum is clearly contaminated by the polymer, with the sharp peaks typical of organic group signals that characterize many polymeric materials. The thickness of the flake allowed the electromagnetic radiation to reach the opposite surface of the sample.

In the blue spectrum, the broad peaks are indicative of an amorphous material. The wide peak visible between 3500 and 3000 cm^−1^ is typical of the -OH group, with the stretching of the -OH bond generating such a broad peak [13], and confirms the presence of hydroxides that could be responsible for the important amorphous phase observed in the XRD analyses. A small peak at 875 cm^−1^ could correspond to the presence of MoO_3_ [14], and, in particular, to the stretching movements of Mo=O group [15,16]. Another type of bonding between Mo and O could be responsible of the peak visible at 600 cm^−1^; this peak could represent the bending mode of Mo-O-Mo. Guzman et al. revealed that a peak near 1615 cm^−1^ could be related to the bending vibration mode of the coordinated water molecules in MoO_3_·nH_2_O [16]. However, some peaks could manifest the presence of cuprite (Cu_2_O), such as the one at around 621 cm^−1^ [17]. The presence of copper compounds is clearly possible since fragments of substrate material might be removed during the sampling process.

### 3.4. Gamma Spectrometry

**Figure 13:**
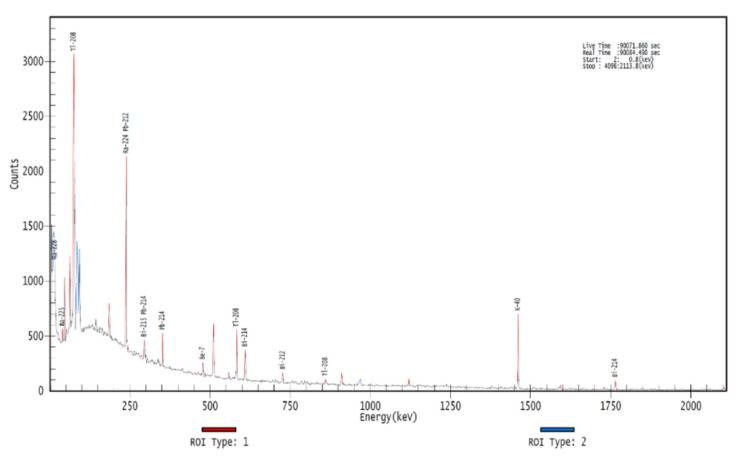
Gamma spectroscopy on BF probes.

The gamma spectroscopy conducted on the BF probes revealed that no activated atoms were present; the obtained spectra are analogous to the environmental one. At 105 and 1205 keV, 91Nb produces characteristic peaks [18], with no activated species seen in the spectrum (Figure 13). Thus, the Nb detected by SEM-EDS could be just an artifact, since the Nb and Mo EDS peaks are very close to each other, or the element could have already been inside the bulk Mo as an impurity. Another hypothesis is that gamma spectrometry analysis was conducted after the half-life period (61 days), leading to the drastic reduction of the activated atoms, which then do not produce an appreciable signal.

## 4. Conclusions

Samples taken from the lower part of the BP, PG and Plasma Box have been examined with spectroscopy and microscopy techniques, with none of them displaying the presence of caesium or its compounds. This is likely due to a water leak in one of the drivers, which might have washed away all the Cs distributed on the surface of the source components. Cs was only found on BF probes that were fastened in the upper part of the SPIDER source, with this being the only experimental evidence of the appearance of the Cs layer distributed on the beam source during the last experimental campaign. The BF probe positioned at the bottom of the source (BF41) confirmed the absence of Cs due to water action. Cs was not dispersed homogeneously on the surface; the expected Cs monolayer, the optimal configuration for surface negative ion production, could not be detected since Cs was found distributed in small agglomerates. The dimensions of such agglomerates were extremely variable, but they all had a circular indented structure. In addition, mapped areas highlighted that oxygen is clearly concentrated in correspondence with Cs: the hypothesis is that the caesium was oxidized after exposing the BP to the atmosphere on opening the SPIDER source. Ti-Cs porous chips, found on the BF probes surfaces, are fragments of Cs-ovens nozzles that have been distributed around the source. The nozzles should be studied to confirm this statement. Further study is needed to understand if the agglomerates were already present before the shutdown and the interactions between Cs and water. Additional investigations of other SPIDER components exposed to Cs evaporation during SPIDER operation are required to analyze the chemical composition, Cs dispersion and the efficient formation of a thin layer. A test bed is under design and construction at Consorzio RFX to study the caesium monolayer distribution on SPIDER grids.

## Figures and Tables

**Table 1:** Samples taken from SPIDER source, the identification code reminds the original location (PDP, BP and PG), except for Sample 3, which is a mixture of several compounds.

Sample Number	Identification Code	Location	Aspect	Type of Analyses
n.3	3	PDP, BP, PG	Mix of colours: green and white	FTIR
n.4	PDP_4	PDP	White compound, flake shape	SEM-EDS
n.6	PDP_6	PDP	White compound	SEM-EDS
n.9	BP_9	BP bottom left	Greenish compound	SEM-EDS, XRD, XPS
n.10	BP_10	BP bottom right	White compound	SEM-EDS, XRD
n.11	PG_11	PG beamlets	Molybdenum chips	SEM-EDS, XRD, XPS

**Table 2:** Chemical composition percentage.

	Cu at. %	Mo at. %	Mo/Cu
Sample BP_9	33.3	66.7	2.0
Sample PG_11	47.9	52.1	1.1
